# Quantitative and Qualitative Genetic Studies of Some Acacia Species Grown in Egypt

**DOI:** 10.3390/plants9020243

**Published:** 2020-02-13

**Authors:** Nader R. Abdelsalam, Hayssam M. Ali, Mohamed Z. M. Salem, Hosam E. El-Wakil

**Affiliations:** 1 Agricultural Botany Department, Faculty of Agriculture (Saba Basha), Alexandria University, Alexandria 21531, Egypt; hosamelwakil@gmail.com; 2 Botany and Microbiology Department, College of Science, King Saud University, P.O. Box 2455, Riyadh 11451, Saudi Arabia; hayhassan@ksu.edu.sa; 3 Timber Trees Research Department, Sabahia Horticulture Research Station, Horticulture Research Institute, Agriculture Research Center, Alexandria 21526, Egypt; 4 Forestry and Wood Technology Department, Faculty of Agriculture (EL-Shatby), Alexandria University, Alexandria 21545, Egypt

**Keywords:** acacia species, isozyme, morphology, plant genetic, genetic differentiation, RAPD-PCR, qualitative characteristics

## Abstract

The objective of the current work is to study the genetic differentiation between *Acacia* species growing in Egypt as plant genetic resources based on morphological, biochemical, and molecular markers. The 20 replicates of *Acacia* tree collected from four localities from Egypt were *A. tortilis* ssp. *raddiana* and *A. farnesiana* (Siwa Oasis and Borg El-Arab City), *A. stenophylla*, *A. sclerosperma* (Marsa Matroh City), and *A. saligna* (Abis Station Farm, Alexandria). The results based on the previous markers indicated highly significant differences between *Acacia* species, confirming the hypothesis of the possibility of using morphological, biochemical, and molecular parameters in species identification. Qualitative characteristics results indicated some similarities and differences that are taxonomically important for comparing taxonomical grouping with morphological data for the genetic description of *Acacia* species. The activities of antioxidant enzymes have been studied intensively and the results provide strong similarities between the *Acacia* species (69%), between *A. raddiana* (Siwa and Borg Al-Arab) and *A. saligna*, followed by all *Acacia* species (50%). Finally, the molecular studies showed that a total of 563 amplification fragments, 190 fragments were monomorphic, and 373 fragments were polymorphic. The highest number of amplification fragments (21) was detected with OPB-20 primer, while OPA-20 showed seven amplification fragments; the average number was 13.09. The results indicated that *Acacia* species exhibit high genetic differentiation, helpful in the future for genetic improvement programs. The novelty of the current study is highlighting the importance of plant genetic resources in Egypt and using different techniques to measure the differentiation between these species.

## 1. Introduction

Egypt is well known for its expansive desert, as approximately 80% of the country is desert. This desert is largely composed of sand dunes, sheets, bed rock, sandstone, limestone, and salt marshes. In the western desert of Egypt, there are a series of oases stretching longitudinally from the north to the far south in a line almost parallel to the Nile valley. This represents natural depressions rich in natural springs and wells that can be used for agriculture. One of the advantages of this desert is the presence of wild flora adapted to biotic stresses such as high temperature, drought, and salinity. Despite the rarity of wild plant cover in such regions, the existing flora is a trove of genetic resources. Most of these floral species are considered the ancestors of domesticated plants, while others possess medicinal benefits, either at a popular level or as documented medicinal treatments. *Acacia tortilis* ssp. *raddiana* is growing in Siwa Oasis as wild flora. By the end of the 21st century, the plant life in the oases of Egypt will have completely changed; about 500,000 acres are expected to be reclaimed and cultivated after redirecting the Nile water to these areas through the Toschka canal from Lake Nasser (south of Aswan on the Nile Valley) to the Kharga Oasis, and ending in the Farafra Oasis in the Western Desert. The importance of plant conservation within and beyond protected areas and the impacts of global change on protected areas and on species conservation have been shown [1]. Albrecht and Long [2] worked on the legume plants and showed the habitat suitability and herbivores determination of legume species. Shaw [3] showed the importance of diversity in plants and species diversity in restoration plantings, in addition to the other important factors to increase the genetic diversity of threatened tree species 

*Acacia* trees constitute much of the woody vegetation plant communities [4]. In addition to their environmental values regarding soil fixation and fertility, considerable research has focused on the silviculture of *Acacia* species, due to their wide distribution in arid regions and various uses, including fodder, fuel, and medicine [5].

*Acacia* is the second-largest genus in the family Leguminosae, with nearly 1350 species [6,7]. The current classification of *Acacia* consists of three subgenera [7]: *Acacia*, *Aculeiferum* and *Heterophyllum* (=*Phyllodineae*). The base chromosome number in the genus *Acacia* is 26 (x = 13), with polyploidy occurring in several species [8,9]. Vivi Tackholm [10] reported that *Acacia* consisted of 11 different species in Egypt, the most well-known being *A. nilotica* L. species *A. mellifera*, *A. laeta*, *A. glaucophylla*, *A. albida*, *A. tortilis*, *A. raddiana*, *A. nubica*, *A. seyal*, *A. flave*, and *A. etbaice*. These species occupy vast areas of the Nile Valley and Nile Delta. 

Previous studies on *Acacia* seeds suggested that *Acacia* species are a possible source of protein for humans [11]. Acacias can produce crude protein per hectare more than many grain crops. For example, the protein content of *A. mellifera* (41.6%) is close to that of soybean (42.8%) [12].

Peroxidase iso-enzyme assay was determined to be the most appropriate technique for the evaluation of the assessed wild and domesticated acacia. Classified peroxidase patterns were ascribed to different phenotypes. As conventional symbols in electrophoresis analysis, a pattern was first described in terms of anodal (A) or cathodal (C) zones according to their direction of mobility under electrophoresis. A study of genetic polymorphism in the peroxidase enzyme system was conducted on the *Acacia* plants [13].

The activities of antioxidant enzymes have been studied intensively. However, the significance of these enzymes in salt tolerance is still a matter of controversy, as high-antioxidant enzymatic activities have been associated with salt tolerance as well as salt sensitivity. This led to the suggestion that genetic differences in salt tolerance among plants are not necessarily due to differences in their ability to detoxify ROS_4_, despite the large number of studies that correlate efficient antioxidative defense with salt tolerance [14]. Isozyme loci have been used as markers in many genetic studies, such as those on genetic diversity in *Brassica juncea* [15] and seed coat color [16]. Peroxidases are enzymes related to polymer synthesis in the cell wall [17] and play a role in the prevention of oxidative damage caused by environmental stress to membrane-bound lipids [18]. Wisal et al. [19] estimated the intra- and inter-specific genetic variability between four species of Family Fabaceae. They used morphometric and protein profiling to detect the variations between these different species.

Plant peroxidases have been used as biochemical markers for various types of biotic and abiotic stresses due to their role in critical physiological processes, such as the control of growth by lignification, crosslinking of pectins and structural proteins in the cell wall, and catabolism of auxins [20]. Catalases and superoxide dismutases are the most efficient antioxidant enzymes [21]. The morphological and genetic diversities among *A. aroma*, *A. macracantha*, *A. caven*, and *A. furcatispina* were studied using morphometric, isozymal, and random amplified polymorphic DNA (RAPD) approaches by Casiva et al. [22]. The analysis of seven isozyme systems revealed 21 loci, and RAPD analysis showed 34 loci. Most of these loci allowed us to differentiate species, except for *A. aroma* and *A. macracantha*, the two most similar species. The levels of genetic variability estimated by isozymes were higher than those determined from RAPD analyses. Morphometric characteristics were highly significantly different among the species, although *A. aroma* and *A. macracantha* were differentiated only by thorn length.

Proline is an amino acid and compatible solute that commonly accumulates in many plants exposed to various stress conditions, such as salinity. When a plant is subjected to stressful conditions, proline is synthesized from glutamate due to the loss of feedback regulation in the proline biosynthetic pathway [23]. The measurement of proline accumulation is an important criterion for determining plant tolerance to salt stress [24]. In salt-stressed plants, the osmotic potential of the vacuoles is reduced by proline accumulation [25]. It is believed that proline accumulation under environmental stress does not inhibit biochemical reactions and plays a protective role during osmotic stress [25]. It is suggested that low osmotic potential may cause proline accumulation in tissues [26].

Modern tools in molecular biology provide detailed information about the genetic structure of natural populations, which was previously not available. During domestication, genetic variation in crop plants decreased due to continuous selection pressure for traits such as great yield or disease resistance. It is necessary to compare the genetic composition of the germplasm of existing cultivars to their ancestors and related species. Genetic differentiation among populations is principally a function of gene flow via pollen and seed dispersal. Several authors have studied the taxonomy of *Acacia* using morphological characteristics [27]. In the last decade, some studies have alternatively used biochemical and molecular markers [28]. Biochemical and molecular studies have been conducted on African and Australian *Acacia* species to provide markers useful for plant breeding and conservation programs [29]. Isozyme electrophoresis and random amplified polymorphic DNA (RAPD) analysis are broadly used in plant population genetic studies [30]. RAPD has primarily enabled the resolution of complex taxonomic relationships [31]. Additionally, phylogenetic diversity is recognized as a relevant criterion for the conservation of species [32]. Analysis of genetic variation in different plant species was carried out by Attia and Al-Sodany [33], who studied the ecological distribution, and, in addition, genetic variations, of some Aloe species based on RAPD and SSR markers. Asaf et al. [34] detected the complete chloroplast genomes of *Vachellia nilotica* and *Senegalia senegal* and they recorded that this may help to elucidate the genome architecture of these species and evaluate the genetic diversity among species.

*Acacia* species are useful as livestock fodder [35]—Bedouin use *Acacia* leaves and pods for fuel, fodder, and medicine—but there are issues with the identification of species. Genetic descriptions and phylogenetic relationships among *Acacia* species are particularly valuable for the conservation management of evolutionarily diverse flora [36]. Genetic differentiations in plant growing in Egypt were studied based on horticulture, molecular and morphological markers [37]. Genetic variation and differentiation in many plants species were studied based on morphological, biochemical, and molecular markers such as tea (*Camellia sinensis*), and revealed by RAPD and AFLP variation by Wachira et al. [38]; *Cornus florida* L using Isozyme and morphological variation [39]; Cornus [40]; genetic markers and horticultural germplasm management [41]; genetic diversity of Salvia species [42]; micropropagation of *A. chundra* (Roxb.) [43]; genetic diversity and differentiation of invasive *A. longifolia* in Portugal [44]; microsatellite markers used for *A. senegal* [45]; phylogenetic analysis based on nuclear DNA and morphology definitions of eastern Australian species of *Acacia* [46]; phylogenetic connections of phyllodinous species of *Acacia* outside Australia [47]; assessment of the phenology of *A. longifolia* [48]; characterization of microsatellite markers used for tree legume *A. koa* [49]; genetic diversity of Australian acacias [50]; genetic diversity of *A. senegal* (L.) willd in Kenyan populations combined RAPD and ISSR markers [51]; genetic diversity and structure of *A. senegal* (L.) Willd. in Uganda [52,53]; molecular phylogeny of *Acacia* s.s. [54]; genetic consequences of anthropogenic disturbances and population fragmentation in *A. senegal* [55]; isolation and characterization of SSR markers in the East African tree, *A. brevispica* [56], and genetic variation in natural populations of *A. visco* [57]. Taylor and Dhileepan [58] showed the effect of the change in phylogenetic relationships of *Acacia* species on the biological control of *Vachellia nilotica* ssp. Also, Monks et al. [59] studied the recovery of some threatened plant species and their habitats in the biodiversity hotspot of the Southwest Australian Floristic Region.

The present study investigated the genetic description and phylogeny of some Egyptian *Acacia* species and subspecies by calculating the morphological, quantitative, and qualitative variations among the species, detecting the biochemical markers based on peroxidase activity and proline content, and estimating the level of polymorphism using RAPD-PCR markers.

## 2. Results and Discussion

### 2.1. Morphological Variations of Acacia Species 

**Figure 1:**
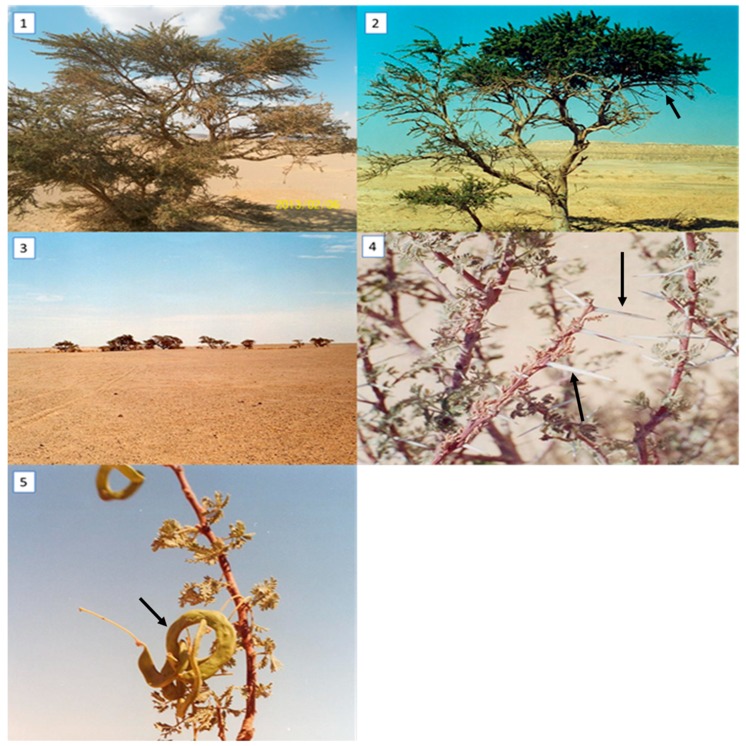
Morphological description of *Acacia* species growing in Egypt (1–5*) Acacia tortilis ssp. raddiana* (collected from Siwa Oasis, an urban oasis in Egypt between the Qattara Depression and the Great Sand Sea in the Western Desert nearly 50 km east of the Libyan border and 650 km from Cairo, 29°12′19 N 25°31’10 E 29°12′19 N 25°31′10 E). (1–3) showing the growth stage of *Acacia* under the desert conditions and (2, 4 and 5) arrows point to the different pine and pinna and show the difference in leaf length, pinna length and spine length. Photos were taken by Coauthor Nader R. Abdelsalam.

**Figure 2:**
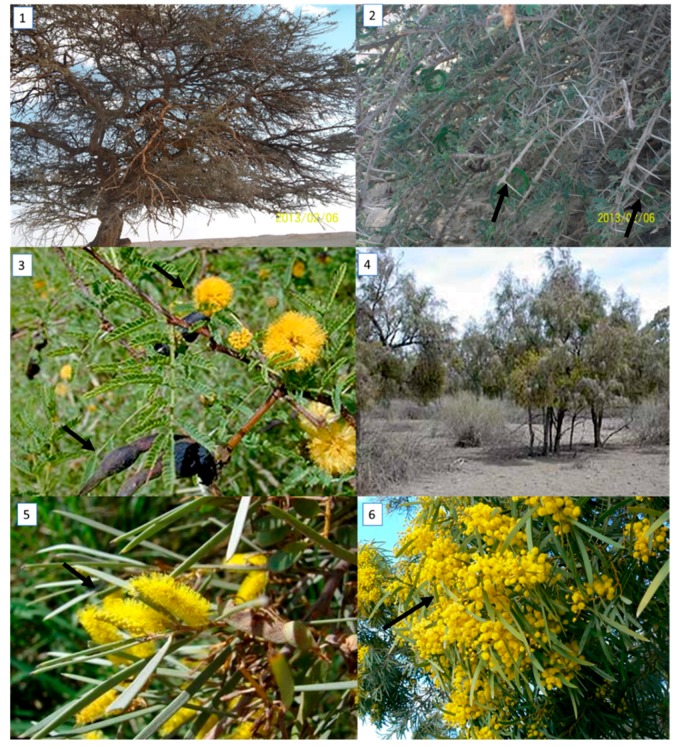
Morphological variations of *Acacia* species growing in Egypt, i.e., (1–2) *A. tortilis ssp. raddiana* (Borg Al-Arab, 48 km south-west of central Alexandria and 7 km Mediterranean Coast, 30°50′56 N 29°36′42 E), arrows point to the different pine and pinna and show the difference in tree form and leaf length, pinna length and spine length, (3) *A. stenophylla* (Marsa Matroh City, 240 km west of Alexandria and 222 km from Sallum on the main highway from the Nil Delta, 31°30′20 N 27°13′13 E), (4) *A. farnesiana* (Borg El-Arab city), (5) *A. sclerosperma* (Marsa Matroh City), and (6) *A. saligna* (Abis Station Farm, Alexandria, 31°12 N 29°55 E) showing the flowering stage. Photos were taken by Coauthor Nader R. Abdelsalam.

The morphological variations among five *Acacia* species (*A. farnesiana, A. tortilis* ssp. *raddiana, A. saligna*, *A. sclerosperma*, and *A. stenophylla*) were determined, and the results are illustrated in Table 1. Some morphological differentiations were also recorded in Figure 1 and Figure 2.

Regarding these parameters, ANOVA revealed very significant differences among the *Acacia* species, confirming the hypothesis of the possibility of identification from such vegetative characteristics. Concerning spine length (mm), data in Table 1 clearly indicate highly significant variation among the different species. We recorded the longest spines in *A. tortilis* ssp. *raddiana* (collected from Siwa Oasis), followed by *A. tortilis* ssp. *raddiana* (Borg Al-Arab city), with means of 28.7 and 19.3 mm, respectively. The shortest spines were found in *A. farnesiana*, i.e., 6.5 mm. No spines were present in the other three species; *A. saligna*, *A. sclerosperma*, and *A. stenophylla* had no spines. 

The results in Table 1 demonstrate an inverse relationship between the spine and pinna length, especially in the desert localities, where these species grow under very unfavorable conditions of drought and high salinity. *A. tortilis* ssp. *raddiana* collected from Siwa and Borg Al-Arab had the shortest pinna length (0.68 and 0.98 cm, respectively) and *A. farnesiana* had the longest pinna length (4.3 cm). As per the phyllode length (cm) data in Table 1, *A. sclerosperma* and *A. saligna* had the longest mean leaf length values of 19.3 cm and 26.1 cm, respectively. The mean shortest leaf lengths recorded were 2.85 cm for *A. tortilis* ssp. *raddiana* collected from (Siwa), 3.2 cm for *A. tortilis* ssp. *raddiana* collected from Borg Al-Arab, and finally 3.80 cm for *A. farnesiana*. The means ranged from 2.0 to 2.5 mm for *A. tortilis ssp. raddiana* (collected from Siwa Oasis = 2 mm), followed by *A. tortilis* ssp. *raddiana* (collected from Borg Al-Arab = 2.50) and *A. farnesiana* (2.25 mm).

Based on the morphological characterization (via qualitative description) we found some similarities and differences that are taxonomically important for comparing taxonomical grouping with morphological data for the genetic description of *Acacia* species. Based on the qualitative results, there were high similarities between *A. farnesiana* and *A. tortilis* ssp. *raddiana*, as both species possessed pinnately compound leaves. On the other hand, *A. saligna, A. sclerosperma*, and *A. stenophylla* had simple leaves (Table 2). The same trend was observed for the growth type, with shrub/small trees being compared to other species that were shrubs or trees. The highest number of stems was recorded for *A. farnesiana* (2–5 stems), followed by both *A. tortilis* ssp. (1–4 stems) and *A. stenophylla* (typically 1 stem). Concerning spine shape, the spines were small in *A. farnesiana* but were long, white, and straight in both *A. tortilis* ssp., as shown in Figure 1 and Figure 2.

Morphological studies were conducted on *Acacia* species in different Arabian countries, such as Morocco, by El Ayadi et al. [60]. In their study, the authors assessed the variability in eight pod traits of 300 genotypes (mother-tree) of *A. tortilis* ssp. *raddiana* (Savi) ‘Brenan’, collected from the southern regions of Morocco. The ANOVA results showed that *A. raddiana* exhibited significant differences in traits due to genotypes within provenances, i.e., in the pod length, seed weight per pod, seed number per pod, infected seed number per pod, and 100-seed weight.

In general, the phenotypic coefficient of variation was higher than the genotypic coefficient of variation, indicating the predominant role of the environment. Their results also demonstrated great heritability and genotypic gain for the 100-seed weight (92.75% and 17.20%, respectively), empty pod weight (53.68% and 21.18%, respectively), and pod weight (46.45% and 16.13%, respectively), indicating additive gene action.

Our results are consistent with those of Quentin et al. [61], who reported that *A. saligna* grows as a small, dense, sprawling tree with a short trunk and a weeping habit. It grows up to 8 m in height. Like many *Acacia* species, it has phyllodes rather than true leaves and these phyllodes can reach a length of 25 cm. 

### 2.2. Biochemical Analysis

#### 2.2.1. Isozyme Assay 

**Figure 3:**
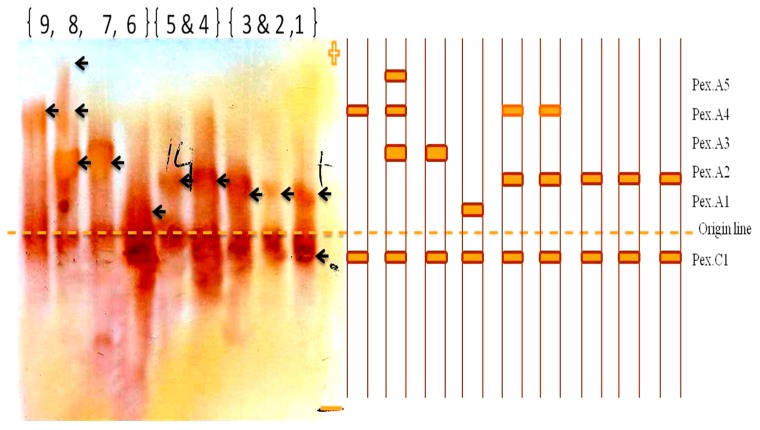
Zymograme of peroxidase isozyme of *Acacia* ssp. (1, 2 and 3) *tortilis ssp. raddiana* (Siwa), (4&5) *tortilis ssp.* *raddiana* (Borg El-Arab), (6) *A. farnesiana*, (7) *A. stenophylla*, (8) *A. sclerosperma* and (9) *A. saligna*.

Peroxidase isozymes exhibited a wide range of variability among the different species at different localities (that ranged in Siwa from deep depression that reaches blew sea level, to about -19 matters, to hot semi-arid climate with moderate temperatures in Alexandria, to hot desert climate in Marsa Matruh). One cathodal (Pex.c1) band was observed as a common band for all samples. The results revealed five anodal (Pex.a1; Pex.a2; Pex.a3, Pex.a4, and Pex.a5) bands recorded for all species. Pex.a1 was recorded in *A. farnesiana* specie, Pex.a2 was recorded in *A. tortilis* ssp., and Pex.a3 was recorded in both *A. stenophylla*, and *A. sclerosperma*, and Pex.a5 was observed in *A. sclerosperma* (Figure 3).

**Figure 4:**
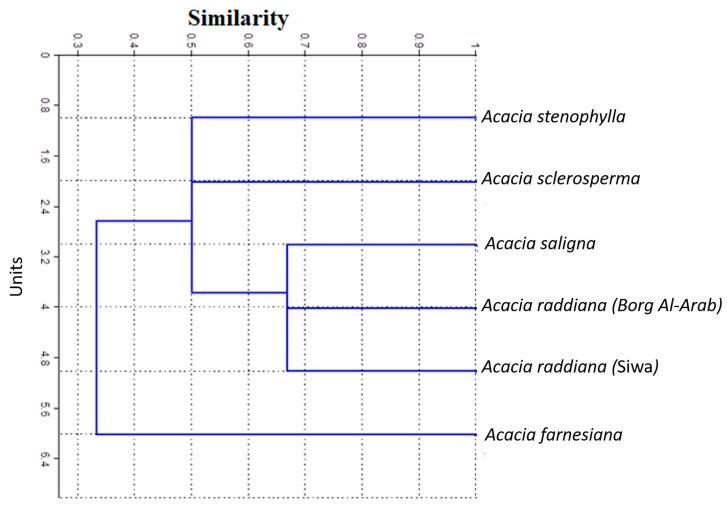
Similarity and genetic distance of *Acacia* spp. Under the certain study.

The data indicate that the peroxidase patterns in the leaves of wild *A. raddiana* (Siwa) and the five domesticated *Acacia* species showed two kinds of banding profiles. First, it was evident that all plants expressed Px.c1, and the five domesticated plants exhibited the same banding profile containing this one locus. This indicated that one common locus was consistently monomorphically expressed. Second, the *A. raddiana* (Siwa) wild types shared one common locus (Px.a1). The banding pattern activity of *Acacia* displayed a unique marker band at the Px.1a and Px.5a loci, indicating that the Px.a2, Px.a3, and Px.a4 loci are specifically polymorphic. The similarity and genetic distance of *Acacia* spp. are presented in Figure 4.

Bands common among all examined species were estimated using average values and were standardized prior to cluster analysis. Data shown in Figure 4 reveal strong similarities among the *Acacia* species located in the first cluster (69%), between *A. raddiana* (Siwa and Borg Al-Arab) and *A. saligna*, followed by all *Acacia* species (50%) in the last cluster (except for *A. farnesiana*), and the similarity between all *Acacia* species was 33%. Salt stress was found to increase the intensity of the peroxidase bands. *A. tortilis* ssp. *raddiana* exhibited greater band intensity compared to the other species. 

#### 2.2.2. Proline Content

The highest content according the Table 3, *A. tortilis ssp. raddiana* (*Siwa Oasis*) had the highest proline content (43.4 μmol/g fresh weight), whereas *A. sclerosperma* had the lowest value (7.6 μmol/g fresh weight) (Table 3). There was highly significant variation among all species in relation to the proline content, and this variation was associated with environmental effects and conditions. *Acacia tortilis* ssp. *raddiana* (Borg Al-Arab) had the highest proline content (23.1 μmol/g fresh weight). The results supported the conclusion that proline accumulates in greater concentrations in plants growing in salty, dry soil and may be useful as a salt injury indicator in plants. This variation in proline could be useful in the selection for salt tolerance and as a marker of salt-tolerant plants. Genotypic variations in proline accumulation have been observed [62], and attempts have been made to correlate its accumulation with stress tolerance in plants. This apparent correlation between proline accumulation and environmental stress suggests that proline might exhibit a protective function [63].

### 2.3. Molecular Markers (RAPD-PCR)

#### Random Amplified Polymorphic DNA (RAPD) Analysis

**Figure 5:**
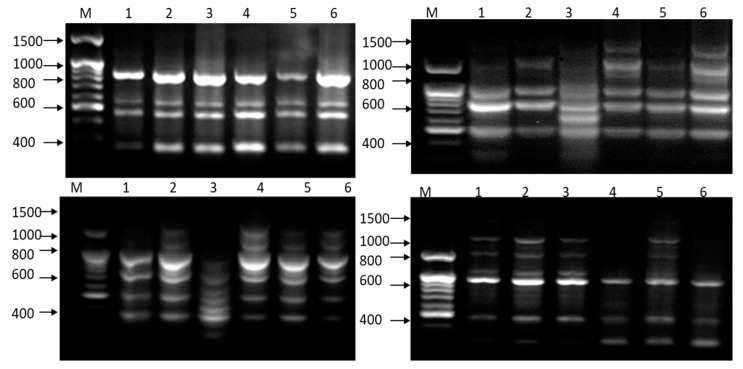
Profile examples of *Acacia* species on agarose gel, amplified with RAPD primers, i.e., OPB-03, OPB-17, OPB-20 and OPQ-12. M = Molecular marker (200: 1500 bp), (1) *A. tortilis* ssp. *raddiana* (Siwa Oasis), (2) *A. tortilis* ssp. *raddiana* (Borg Al-Arab), (3) *A. stenophylla*, (4) *A. farnesiana*, (5) *A. sclerosperma* and (6) *A. saligna*.

In the present study, the genetic variability and relationships of different *Acacia* species were studied based on RAPD analysis. The initial screening of 52 primers with six samples resulted in 43 primers that produced informative and polymorphic products resolvable by agarose gel electrophoresis (Table 4). Profile examples of *Acacia* species on agarose gel, amplified with RAPD primers, are found in (Figure 5).

Out of 563 amplification fragments, 190 fragments were monomorphic, and 373 fragments were polymorphic (Table 4). The maximum number of amplification fragments recorded was 21 for primer OPB-20, compared with the lowest, *i.e.*, seven for primer OPA-20. The average number of amplification fragments was 13.09 for all 43 primers (Table 4). 

Concerning the monomorphic fragments, eight was the highest number of fragments recorded with primers OPB-03 and OPN-04, and one was the lowest number of fragments recorded for primer OPA-20. The general mean of the monomorphic fragments was 4.42 for all primers. Regarding polymorphic fragments, 14 was the highest value, recorded for primers OPA-16, OPB-20 and OPQ-14, while four fragments was the lowest detected value (OPA-09). The average number of polymorphic amplification fragments was 8.67 (Table 4).

**Figure 6:**
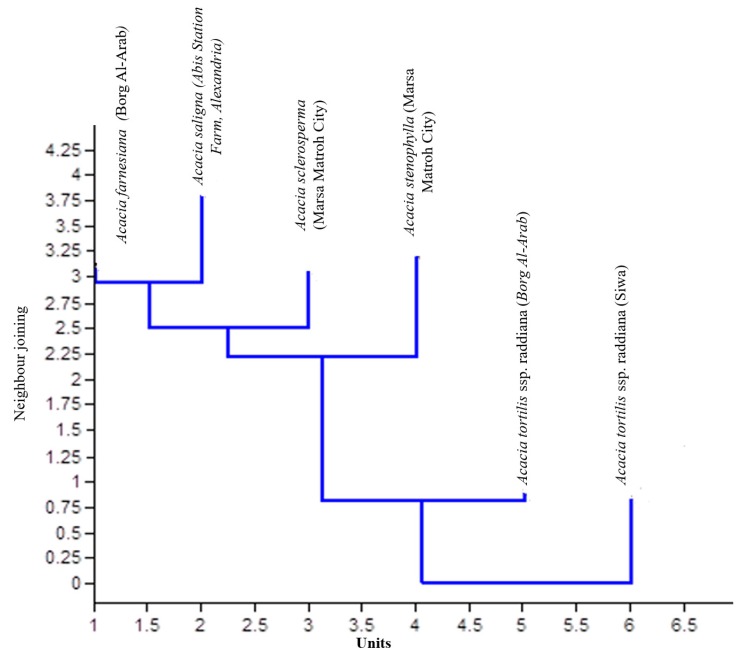
Dendrogram of similarity of different *Acacia* species based on forty-three RAPD primers.

The percent polymorphic loci (PIC%) values ranged from 85.71% for primer OPA-20 to 42.85% for primer OPN-04. The PIC% between the six *Acacia* species using 43 RAPD primers was 66.46% (Table 4 and Figure 6).

### 2.4. Genetic Similarity and Phylogenetic Relationships Among Acacia Species

Genetic similarities and phylogenetic relationships among the six tested samples of *Acacia* species were examined using RAPD-PCR analysis, and the obtained data were subjected to cluster analysis with a Dice equation using SPSS (ver. 15) to calculate the proximity matrix and design dendrogram. Genetic similarity values generated from the RAPD markers varied between 0.60 and 0.78, with an average of 0.69. The dendrogram was based on similarity values (Table 5) from RAPD and was constructed using SPSS (ver. 15) to reveal similarities between the five different *Acacia* species. The dendrogram (Figure 6) demonstrated that the six *Acacia spp.* fell into three main groups. The first group included *A. tortilis* ssp. *raddiana* (Siwa Oasis), the second group included *A. tortilis* ssp. *raddiana* (Borg Al-Arab), and the third group included the other *Acacia* species.

RAPD-PCR markers are used routinely to identify genetic variations [64]. RAPD markers have also been used successfully in various taxonomic and phylogenetic studies by Kazan et al. [65] and Wilkie et al. [66]. As a molecular marker system, RAPD has also been successfully applied in cultivar identification. RAPD analysis is normally easy to perform, but it has a major disadvantage in that reproducibility is difficult to achieve between different laboratories, and often even between different people in the same laboratory. The previous results are consistent with Fagg and Allison [67], who reported variation in the chemical composition as well as molecular and morphological characteristics between Ugandan and Sudanese populations of *A. senegal*. Our results are in line with those of Shrestha et al. [68], who investigated *A. raddiana* populations and reported a great degree of polymorphism contrary to the conventional expectation of small, isolated populations. The maintenance of genetic variation is important because future evolutionary adaptation depends on the existence of genetic variation.

The PIC% values obtained in this study were far higher than those observed in *A. caven* (29.4%) [22], *A. anomala* (43%) [69], and *Faidherbia albida* (42.7%) [70]. However, similar results were obtained for *Haloxylon ammodendron* (74.9%) by Sheng et al. [71] using ISSR markers, for *Changium smyrnioides* (69%) by Fu et al. [72] using RAPD markers, and for *F. albida* (90%) by Joly et al. [73] using isozymes.

## 3. Materials and Methods 

### 3.1. Leaf Samples

Entire leaves of the following species were randomly collected from 20 individuals in natural *Acacia* tree habitats: *A. tortilis* ssp. *raddiana* collected from (Siwa Oasis) is an urban oasis in Egypt between the Qattara Depression and the Great Sand Sea in the Western Desert, nearly 50 km east of Libyan border and 650 km from Cairo, 29°12′19 N 25°31′10 E and (Borg El-Arab City, Alexandria, 48 km south-west of central Alexandria and 7 km from the Mediterranean Coast, 30°50′56 N 29°36′42 E); *A. farnesiana* (Borg El-Arab City)*; A. stenophylla* (Marsa Matroh City, 240 km west of Alexandria and 222 km from Sallum on the main highway from the Nil Delta, 31°30′20 N 27°13′13 E)*, A. sclerosperma* (Marsa Matroh City)*;* and *A. saligna* (Abis Station Farm, Alexandria, 31°12N 29°55E).

### 3.2. Morphological Analysis

Four samples were collected for each parameter. The following morphological parameters were measured in the vegetative parts of the tree: pinna length (cm), leaf length (cm), leaflet length (mm), and spine length (mm) (if present) were measured with a millimetric ruler. Some qualitative characters were also recorded, such as crown shape (round, flat, spread, or undefined), growth habit, and number of stems counted from the ground level (1, 2–5, or > 5). The spine shape observed in individual plants was determined to be either straight or mixed, i.e., straight and curved.

### 3.3. Biochemical Assays

#### 3.3.1. Iso-Enzyme Electrophoresis 

Agar starch polyvinyl pyrolidine (PVP) gel electrophoresis was conducted according to the protocol described by Andrews [13]. The extracts were made by grinding young leaf tissue in a mortar with 10 µL of electrode buffer and centrifuging for 15 s; a 10 µL sample of the homogenate was then absorbed onto a small rectangle (approximately 4 × 2 mm) of filter paper that was placed on the origin line of gel plates, which was removed after storage at 4 °C for 30 min. The buffer was prepared by dissolving 92.75 g of 0.3 M boric acid and 12 g sodium hydroxide in 5 L of distilled water; then, the solution was adjusted to pH 8.3 according to Ahmed [74]. The gel buffer used was 0.07 M Tris 0.007 M citric acid, pH 8.3. One liter of the gel buffer was prepared by dissolving 9.21 g Tris and l.05 g citric acid in distilled water and stored in a refrigerator until use. Ten grams of PVP gel were dissolved.

The mixture was vigorously shaken and cooked in a boiling water bath until the solution was transparent. The hot liquid gel was poured over glass plates (20 × 30 cm) to produce a smooth surface layer with a thickness of 0.8–0.9 mm and stored at 4 °C until use [75]. The electrophoresis experiment was conducted in an incubated refrigerator set to 4 °C using a 250 V AC electrical current with constant voltage during the 90 min running period. A phosphate buffer (0.l M, pH = 7.0) was used as a staining buffer by adding 39 mL of a 0.l M solution of monobasic sodium phosphate to 61 mL of a 0.1 M solution of sodium dibasic phosphate and increasing the final volume to 200 mL using distilled water. Each gel was incubated in l00 mL phosphate buffer, pH 7.0, containing 20 mg *α*-naphthyl acetate (*α*-NA) and 20 mg *β*-naphthyl acetate (NA), dissolved in l mL acetone, and brought to 5 mL using distilled water. Fast blue RR salt (C_15_H_14_ClN_3_O_3_·_1_/_2_ZnCl_2_) (50 mg dissolved in 5 mL distilled water) was added 3 min after the addition of *α*- and *β*-NA. This implies that an additional incubation was carried out for 30 min at 27–30 °C under complete darkness. Plates were then washed with distilled water [76].

#### 3.3.2. Proline Content

Proline was determined according to the method presented by Bates et al. [77]. The mixture was warmed by agitation until dissolved, then kept cool at 4 °C until use. A 0.5 g sample of leaf material was homogenized in 10 mL extraction buffer. The homogenate was filtered through Whatman filter paper No. 2, and 2 mL of filtrate was reacted with 2 mL acid ninhydrin and 2 mL glacial acetic acid in a test tube for 1 h at 100 °C. The reaction was terminated using an ice bath. The reaction mixture was extracted with 4 mL of toluene mixed vigorously in a test tube with a stirrer for 15–20 s. The chromosphere containing toluene was aspirated from the aqueous phase, and the absorbance was determined using a spectrophotometer at 520 nm using toluene as a blank. The proline content was expressed as fresh weight on a standard curve, using standard L-proline according to the previous method developed by Hasan et al. [78]. Briefly, as follows: µmol proline g of fresh plant material = [(µg proline/mL^−^ × mL toluene)/115.5 µg/µmol/(g sample/5)] [63].

### 3.4. Molecular Analysis

#### 3.4.1. Random Amplified Polymorphic DNA (RAPD) Analysis 

Fifty-two random amplified polymorphic DNA (RAPD) primers (10-mer primers) were initially screened using six *Acacia* species to determine the suitability of each primer for this study. Primers were selected for further analysis based on their ability to produce distinct, clearly resolved, and polymorphic amplified products between *Acacia* species. To ensure reproducibility, primers generating no, weak, or complex patterns were discarded [79]. DNA was extracted from 50 mg samples of *Acacia* leaves using the DNeasy^®^ Plant System (Operon Technologies Inc., Alameda, CA). The DNA extraction was modified, i.e., 700 µL of warm (up to 65 °C) buffer AP1 for lyses was required during homogenization. Samples were centrifuged for 30 s at low speed (4000× *g*), and 7 µL of RNase A stock solution (100 mg·mL^−1^) was mixed into each tube until no tissue clumps were visible.

The mixture was incubated for 20 min at 65 °C, and tubes were inverted twice during the incubation period. The addition of 228 µL buffer AP2 to the lysate and incubation on ice for 5 min precipitated the detergent, proteins, and polysaccharides. The column-tube assembly was centrifuged at maximum speed for 2 min in a microcentrifuge. At least 500 µL of clear filtrate was transferred to a 1.5 mL microfuge tube. Buffer AP3 and 100% ethanol (1:2 v/v) were added to the lysate, and the solution was gently mixed by pipetting. Up to 650 µL of the sample mixture (including any precipitate that formed) was applied to the DNeasy (Operon Technologies Inc., Alameda, CA) spin column, which was placed in a 2 mL collection tube. The assembly was centrifuged for 1 min at 12,000× *g*, causing the DNA to be bound to the column membrane, and the filtrate was discarded. The remaining sample mixture was applied to the same column, and the procedure was repeated. The collection tube was replaced by a clean 2 mL tube, and the column was washed two to three times by adding 500 µL buffer AW (containing ethanol) to the DNeasy column, centrifuging for 1 min at 12,000× *g*, and discarding the filtrate.

Following the final wash, the column–tube assembly was centrifuged for 2 min at 12,000× *g* to dry the column membrane, and the collection tube was discarded. The DNeasy column was transferred to a clean 1.5 mL microcentrifuge tube, and DNA was eluted from the membrane by pipetting 100 µL of preheated (65 °C) buffer AE directly onto the DNeasy column membrane. The sample was then incubated for 5 min and centrifuged for 1 min at 12,000× *g* or higher. The elution step was repeated using the same microcentrifuge tube and yielded a final volume of 50 µL DNA solution

#### 3.4.2. Polymerase Chain Reaction (PCR) Conditions 

RAPD analysis was conducted using 10 oligonucleotide primers that were selected from the Operon Kit (Operon Technologies Inc., Alameda, CA). The polymerase chain reaction (PCR) mixture (25 µL) consisted of 0.8 U of *Taq* DNA polymerase, 25 pmol dNTPs, 25 pmol of primer, and 50 ng of genomic DNA. PCR amplification was performed in a Biometra *T1* gradient thermal cycler (Phoretix International, Newcastle upon Tyne, UK) for 40 cycles after initial denaturation for 3 min at 94 °C. Each cycle consisted of denaturation at 94 °C for 1 min, annealing at 36 °C for 1 min, extension at 72 °C for 2 min, and a final extension at 72 °C for 10 min [79]. Amplification products were separated on 1% agarose gels at 100 V for 1.30 h, using 1 × TBE buffer. To detect the ethidium bromide/DNA complex, agarose gels were examined using an ultraviolet transilluminator (302 nm wavelength); subsequently, the lengths of the different DNA fragments were determined. For each sample, the reproducible DNA bands from two runs were scored for their presence or absence. Fragment scoring and lane matching were performed automatically on digital images of the gels using Phoretix 1D Advanced version 4.00 (Phoretix International, Newcastle upon Tyne, UK).

All but the faintest bands were scored, and, where necessary, scores and matches were manually corrected. 

Based on the matrix of genetic similarity values (peroxidase isozymes data) and the dendrogram was generated from PAleontological STatistics (PASTA) program that runs on standard Windows computers and is available free of charge. PAST integrates spreadsheet-type data entry with univariate and multivariate statistics, curve fitting, time series analysis, data plotting, and simple phylogenetic analysis. Many of the functions are specific to paleontology and ecology, and these functions are not found in standard, more extensive, statistical packages. PAST also includes fourteen case studies (data files and exercises), illustrating use of the program for paleontological problems, making it a complete educational package for courses in quantitative methods (http://palaeo-electronica.org). 

### 3.5. Statistical Analysis

Data of the morphological variations of *Acacia* species were statistically analyzed with one-way analysis of variance (ANOVA) using the SAS system [80]. Comparisons among means were measured using LSD_0.05_.

## 4. Conclusions

In the present study, genetic differentiation of some *Acacia* species growing in Egypt based on morphological, biochemical, and molecular markers were measured using twenty replicates of *Acacia* tree, collected from four different localities in Egypt. The results clearly indicated highly significant differences between *Acacia* species for morphological characteristics. In addition, the qualitative characteristics were used to detect the similarities and differences which are important in comparing the taxonomical grouping of Acacia species. RAPD-PCR proved to be a powerful tool for assessing the genetic diversity of several *Acacia* species in Egypt. Morphological parameters revealed highly significant variations among the *Acacia* species, confirming the hypothesis of the possibility of identification from such vegetative characteristics. Study of the genetic differentiation of Acacia species growing in Egypt is considered a primary step to genetic improvement in and documentation of this plant genetic resource in Egypt, especially *Acacia tortilis ssp. Raddiana*, which was collected from the isolated area Siwa Oasis in Egypt.

## Figures and Tables

**Table 1:** Morphological variations of *Acacia* species: spine length, pinna length, leaf length and leaflet length.

Species	Spine Length (mm)	Pinna Length (cm)	Leaf Length (cm)	Leaflet Length (mm)
A. tortilis ssp. raddiana **	28.75 ^a^ ± 0.33	0.68 ^c^ ± 0.01	2.85 ^e^ ± 0.05	2.00 ^a^ ± 0.01
A. tortilis ssp. raddiana ***	19.25 ^b^ ± 0.15	0.98 ^b^ ± 0.01	3.15 ^e^ ± 0.11	2.50 ^a^ ± 0.01
*A. farnesiana*	6.50 ^c^ ± 0.16	4.28 ^a^ ± 0.65	3.80 ^d^ ± 0.08	2.25 ^a^ ± 0.11
*A. saligna*	-	-	23.28 ^b^ ± 1.30	-
*A. sclerosperma*	-	-	26.08 ^a^ ± 1.00	-
*A. stenophylla*	-	-	19.25 ^c^ ± 1.77	-
LSD 0.05	1.36	0.252	0.508	0.705

* Mean followed by the same letter (s) is not significantly different at 0.05 levels. (-) not found, ** *A. tortilis ssp. raddiana* (Siwa Oasis), *** *A. tortilis ssp. raddiana* (Borg Al-Arab).

**Table 2:** Qualitative description of some *Acasia* species in Egypt.

Species	Leaves Type of	Growth Form	Crown Shape	Mean of Stems Number	Spine S Hape
A. tortilis ssp. raddiana *	Pinnately compound	Shrub/small tree	irregular/round	3 ± 0.12	Long white straight
A. tortilis ssp. raddiana **	Pinnately compound	Shrub/small tree	irregular/round	2.5 ± 0.02	Long white straight
*A. farnesiana*	Pinnately compound	Shrub/small tree	often spread	3.5 ± 0.26	small
*A. saligna*	Simple	shrub or tree	spread	1 ± 0.01	-
*A. sclerosperma*	Simple	shrub or tree	spread	1 ± 0.01	-
*A. stenophylla*	Simple	shrub or tree	rounded	1 ± 0.00	-

* A. tortilis ssp. raddiana (Siwa Oasis), ** A. tortilis ssp. raddiana (Borg Al-Arab).

**Table 3:** Proline content (μmol/g fresh weight) in some *Acacia* species.

Species	Proline Content (μmol/g Fresh Weight)
*A. tortilis ssp. raddiana* (Siwa)	43.4 ^a^
*A. tortilis ssp. raddiana* (Borg Al-Arab)	23.1 ^b^
*A. stenophylla*	21.7 ^c^
*A. farnesiana*	13.1 ^d^
*A. saligna*	11.5 ^e^
*A. sclerosperma*	7.6 ^f^
LSD 0.05	0.282

Mean followed by the same letter (s) is not significantly different at 0.05 levels.

**Table 4:** Primer name, sequence, total number of amplified fragments and polymorphic percentage for *Acacia* species based on RAPD analysis.

Premier Number	Primer Name	Sequence 3′---5′	TAF	MF	PF	PIC%	Size (Kbp)
1	OPA-01	5′-CAGGCCCTTC-3′	09	02	07	77.78	0.1–2.9
2	OPA-02	5′-TGCCGAGCTG-3′	09	03	06	66.67	0.2–2.6
3	OPA-05	5′-AGGGGTCTTG-3′	11	07	05	45.45	0.1–2.6
4	OPA-09	5′-GGGTAACGCC-3′	08	04	04	50.00	0.2–2.5
5	OPA-11	5′-CAATCGCCGT-3′	13	05	08	61.54	0.1–3.0
6	OPA-14	5′-TCTGTGCTGG-3′	13	03	10	76.92	0.1–2.8
7	OPA-15	5′-TTCCGAACCC-3′	16	06	10	62.50	0.1–2.7
8	OPA-16	5′-AGCCAGCGAA-3′	19	04	14	73.68	0.3–2.6
9	OPA-18	5′-AGGTGACCGT-3′	09	02	07	77.78	0.1–2.6
10	OPA-20	5′-GTTGCGATCC-3′	07	01	06	85.71	0.1–2.9
11	OPB-03	5′-CATCCCCCTG-3′	18	08	10	55.56	0.2–3.0
12	OPB-07	5′-GAAACGGGTG-3′	16	05	11	68.75	0.2–2.2
13	OPB-17	5′-AGGGAACGAG-3′	18	06	12	66.67	0.2–2.3
14	OPB-20	5′-GGACCCTTAC-3′	21	07	14	66.67	0.2–2.4
15	OPC-02	5′-GTGAGGCGTC-3′	15	05	10	66.67	0.1–2.3
16	OPC-05	5′-GATGACCGCC-3′	10	03	07	70.00	0.1–2.3
17	OPC-12	5′-TGTCATCCCC-3′	11	04	07	63.64	0.1–2.3
18	OPC-16	5′-CACCATCCAG-3′	10	04	06	60.00	0.1–2.3
19	OPD-03	5′-GTCGCCGTCA-3′	13	05	08	61.54	0.3–2.5
20	OPD-04	5′-TCTGGTGAGG-3′	13	04	09	69.23	0.3–2.6
21	OPD-05	5′-TGAGCGGACA-3′	14	04	10	71.43	0.1–2.9
22	OPD-08	5′-GTGTGCCCCA-3′	13	04	09	69.23	0.2–2.9
23	OPD-11	5′-AGCGCCATTG-3′	12	03	09	75.00	0.1–2.5
24	OPE-12	5′-TTATCGCCCC-3′	15	06	09	60.00	0.3–2.4
25	OPG-12	5′-CAGCTCACGA-3′	15	03	12	80.00	0.3–2.6
26	OPH-11	5′-AGCGCCATTG-3′	11	05	06	54.55	0.3–2.9
27	OPN-04	5′-GACCGACCCA-3′	14	08	06	42.86	0.1–2.6
28	OPN-05	5′-ACTGAACGCC-3′	13	06	07	53.85	0.1–2.7
29	OPN-08	5′-ACCTCAGCTC-3′	13	06	07	53.85	0.2–2.7
30	OPN-09	5′-TGCCGGCTTG-3′	12	05	07	58.33	0.1–2.9
31	OPN-10	5′-ACAACTGGGG-3′	15	04	11	73.33	0.2–2.0
32	OPN-11	5′-ACAACTGGGG-3′	14	04	10	71.43	0.1–3.0
33	OPN-13	5′-AGCGTCACTC-3′	10	03	07	70.00	0.2–3.0
34	OPN-14	5′-TCGTGCGGGT-3′	10	02	08	80.00	0.2–2.9
35	OPN-15	5′-CAGCGACTGT-3′	10	02	08	80.00	0.1–2.8
36	OPN-16	5′-AAGCGACCTG-3′	09	02	07	77.78	0.1–2.7
37	OPN-17	5′-AGCGTCACTC-3′	11	05	06	54.55	0.1–2.7
38	OPM-05	5′-GGGAACGTGT-3′	14	04	10	71.43	0.1–2.7
39	OPQ-12	5′-AGTAGGGCAC-3′	19	07	12	63.16	0.3–2.6
40	OPQ-14	5′-GGACGCTTCA-3′	17	03	14	82.35	0.1–2.9
41	OPR-01	5′-CTTCCGCAGT-3′	16	05	11	68.75	0.3–2.5
42	OPR-02	5′-GGTGCGGGAA-3′	14	07	07	50.00	0.3–2.5
43	OPR-03	5′-GACCTAGTGG-3′	13	04	09	69.23	0.3–2.9
Total			563	190	373	66.46	

TAF = Total amplified fragments, MF: monomorphic fragments, PF: polymorphic fragments, PIC (%): percentages of polymorphism and Sm: specific marker fragments.

**Table 5:** Similarity indices (%) among *Acacia* species based on 43 random amplified polymorphic DNA (RAPD) primers.

Species	*A. Tortilis* (Siwa)	*A. Tortilis* Borg	*A. Farnesiana*	*A. Stenophylla*	*A. Sclerosperma*	*A. Saligna*
*A. tortilis* (Siwa)	1.00					
*A. tortilis* (Borg)	0.78	1.00				
*A. farnesiana*	0.74	0.71	1.00			
*A. stenophylla*	0.67	0.76	0.60	1.00		
*A. sclerosperma*	0.76	0.73	0.62	0.69	1.00	
*A. saligna*	0.67	0.72	0.60	0.77	0.77	1.00
